# Bilateral Metastatic Gynecomastia from Small-Cell Lung Tumors in a Man: A Report of a Rare Case

**DOI:** 10.5334/jbsr.3596

**Published:** 2024-05-15

**Authors:** Karima El Houari, Sophie Vandewalle

**Affiliations:** 1 Hôpital Universitaire de Bruxelles (H.U.B)-Hôpital Erasme, Belgium; 2 Centre Hospitalier Universitaire Tivoli (C.H.U Tivoli), La Louvière, Belgium

**Keywords:** Gynecomastia, breast metastasis, male, small-cell carcinoma, immunohistochemistry

## Abstract

A case of a man with the recent onset of painful bilateral firm gynecomastia is reported. Mammography confirmed increased breast density. Biopsy characterized both masses as metastases of a small-cell lung tumor.

This case highlights the atypical presentation and complements the literature regarding the rarity of breast metastases from small-cell lung cancer in men.

*Teaching point:* Bilateral gynecomastia in a man with a long history of cigarette smoking should be considered with caution.

## Introduction

Breast metastases from small-cell lung cancer (SLCS) are exceptional in men (0.2%–1.3%) [1, 2]. Differentiating between a primary small-cell breast tumor and breast metastases of a small-cell lung tumor is a diagnostic challenge given their common morphological characteristics [1, 3]. This rare case complements the existing scarce literature and highlights the atypical presentation of small-cell lung tumors in males [4].

## Case Report

We report the case of a 53-year-old man referred for the exploration of bilateral, painful, and firm breast development, which appeared within 1 month. The patient was known for alcohol abuse and was a long-time smoker. There was no personal or family history of breast cancer. A clinical examination confirmed bilateral gynecomastia with induration.

**Figure 1:**
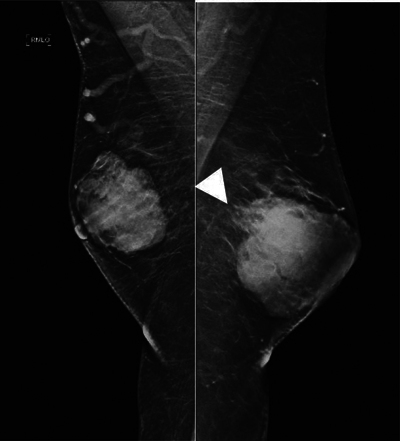
Mammogram with external oblique view of the right breast and the left breast demonstrating an increase in breast density with a macrolobulated shape bilaterally, with blurred posterior contours (white arrowhead) and retronipple topography. No contact with the pectoral muscle.

A mammogram revealed the presence of a macro-lobulated retro-nipple opacity bilaterally (Figure 1), measuring 52 × 38 × 50 mm on the right and 50 × 50 × 40 mm on the left, and with blurred posterior contour (white arrow). Both masses were classified as BIRADS 4 (Figure 1).

**Figure 2:**
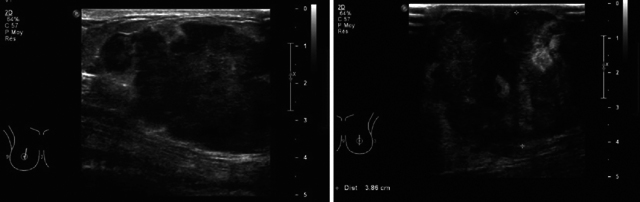
**(a)** Ultrasound right breast longitudinally. **(b)** Ultrasound the left breast. Ultrasound confirmed the presence of an irregular marginated lobulated hypoechogenic and vascularized masses in both breasts.

Ultrasound confirmed the presence of an irregular marginated lobulated hypoechogenic and vascularized masses in both breasts (right Figure 2a; left Figure 2b). Bilateral axillary lymphadenopathy with cortical thickening was also present.

Microbiopsies of the breast masses and cytopuncture of axillary lymphadenopathy revealed breast metastases of a small-cell tumor.

**Figure 3:**
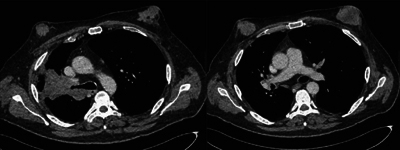
Thoraco-abdominal CT scan injected into the parenchymal window: left upper lobar mass stenosing the right lobar bronchus and “mass” type breast tissue development, bilaterally.

The thoraco-abdominal computed tomography (CT) scan confirmed the presence of a large right upper lobar budding tumor, histologically a small-cell bronchial carcinoma (Figure 3).

The treatment proposed was chemotherapy and immunotherapy.

## Discussion

SLCS is an aggressive neuroendocrine tumor that exceptionally metastasizes to the breast (0.2%–1.3%) [1, 5]. Pulmonary SLCS is often diagnosed incidentally, given its insidious nature [2, 6]. There are only two cases of bilateral breast metastases from pulmonary SLCS in male patients reported in the literature in 1976 and 2011 [7, 3].

Malignancy must be considered in cases of recent-onset gynecomastia associated with smoking [8]. In addition, it is imperative to distinguish between a primary breast and metastasized pulmonary SLCS because the therapeutic approach and the prognosis are different [3, 9, 10].

Although breast metastatic lesions are more superficial and do not cause skin retraction, a distinction between both entities is impossible [1, 11]. A biopsy is required and the final diagnosis is based on the histological and immunohistochemical analysis, including TTF1 marker, neuroendocrine (NSE, chromogranin A, and synaptophysin), and hormonal receptors [3, 10]. The TTF-1 marker not being specific on its own. [1].

## Conclusion

Breast metastases from SLCS are exceptional, especially in male. The distinction between pulmonary and breast small-cell tumors is a diagnostic challenge in imaging. Biopsy and extensive immunohistochemical analyzes are essential for differentiation and therapeutic management.
